# Robotic treatment of oligometastatic kidney tumor with synchronous pancreatic metastasis: case report and review of the literature

**DOI:** 10.1186/s12893-018-0371-x

**Published:** 2018-06-13

**Authors:** Andrea Boni, Giovanni Cochetti, Stefano Ascani, Michele Del Zingaro, Francesca Quadrini, Alessio Paladini, Diego Cocca, Ettore Mearini

**Affiliations:** 1 Department of Surgical and Biomedical Sciences, Division of Urological, Andrological surgery and Minimally-invasive techniques, University of Perugia, Perugia, Italy; 2 Institute of Pathologic Anatomy, “Santa Maria” Hospital, Terni, Italy

**Keywords:** Metastasectomy, Kidney cancer, Distal atypical pancreasectomy, Spleen-preservation, Robot-assisted surgery

## Abstract

**Background:**

The management of metastatic Renal Cell Carcinoma (RCC) has changed dramatically in the last 20 years, and the role of surgery in the immunotherapy’s era is under debate. Metastatic lesions interesting pancreas are infrequent, but those harbouring from RCC have an high incidence. If metachronous resections are not rare, synchronous resection of primary RCC and its pancreatic metastasis is uncommonly reported, and accounts for a bad prognosis.

**Case presentation:**

We report the case of a 68 years old woman, who presented hematuria at hospital incoming, with radiological appearance of a 13 cm left renal mass, with a 2.5 cm single pancreatic tail metastasis. Work-up of staging ruled out other distant metastases, urothelial cancer and there was no evidence of inferior vena cava thrombosis. We choose a 5-port trans-peritoneal robotic approach using lazy right lateral decubitus. Synchronous robotic radical nephrectomy and spleen-sparing pancreatic resection was performed. The pancreatic mass was completely enucleated from pancreatic parenchyma using a latero-medial dissection. Peri-operative hemoglobine loss was 2.4 g/dL. Total operative time was 213 min. No post-operative complications were recorded and patient was discharged in 7th post-operative day. Histopathological examination showed a pT2b N0 M1 RCC, Fuhrman grade II, with pancreatic tail metastasis; both, primary and metastatic lesions had the same histological characteristics with negative surgical margins. After 9 months patient had no evidence of disease recurrence at radiological studies.

**Conclusions:**

The rationale for surgical removal of disseminated tumor, followed by immunotherapy, includes improving prognosis and enhancing the potential of an immune-mediated response to systemic treatment. A spleen-sparing procedure can adequately preserve post-operative immunologic capabilities. In our experience, the correct assessment of pre-operative imaging data and surgeon skills in robotic surgery seem to play a key role in the success of these procedures. Robotic surgery seems to enhance the possibility to control multiple vessels encountered during dissection. Such a conservative approach may be helpful in future research aimed at uncovering biological features, and also leading to better targeted preventive interventions and more individualized and effective treatments.

**Electronic supplementary material:**

The online version of this article (10.1186/s12893-018-0371-x) contains supplementary material, which is available to authorized users.

## Background

Renal Cell Carcinoma (RCC) represents 2–3% of all adult neoplasms. It is the prevalent type of kidney cancer, accounting for a broad spectrum of histological entities. The three most represented RCC types are: clear cell, papillary and chromophobe [1, 2]. Unfortunately, more than 20% of patients are diagnosed with metastasis at clinical presentation. The association with locally advanced RCC worsen the prognosis [3]. In 75% of cases metastases are hematogenous and spread through the renal vein and the vena cava towards lungs, liver, adrenal glands and, skin with the pancreas fifth frequently involved organ [4]. In fact, RCC represents the most common primary tumour leading to pancreatic metastasis, that accounts for at least 2% of all pancreatic malignancies [5, 6].

Metachronous resection of metastases from primary RCC are more commonly described than synchronous one and time of metastatic onset is discussed as an important prognostic factor [7, 8]. To our knowledge, only four studies reported synchronous treatment of RCC pancreatic metastasis, using “en bloc” removal of kidney, spleen and pancreatic tail [9–11]. However, in advanced renal disease the role of surgery is debated mainly because of significant post-operative morbidity, beyond the development of new immunotherapies [11, 12]. Moreover, the pancreatic metastasectomy should be performed on a patient with good performance status and at an experienced center, when a survival benefit could be proven [3, 13]. Both laparoscopic and robotic approaches have been established as safe and seem to have comparable outcomes for pancreatic surgery, although the last one may be associated with fewer conversions rate and a more intuitive approach [14, 15]. The first case of robot-assisted “en bloc” radical nephrectomy, splenectomy and distal pancreatectomy, for a locally advanced RCC, was only recently reported [16] .

Herein, we present a synchronous robot-assisted treatment of an oligo-metastatic kidney cancer with a pancreatic tail metastasis. To our knowledge, this is the first report of a simultaneous robotic treatment of a kidney cancer with resection of its pancreatic metastasis, without removal of the spleen.

## Case presentation

**Fig. 1:**
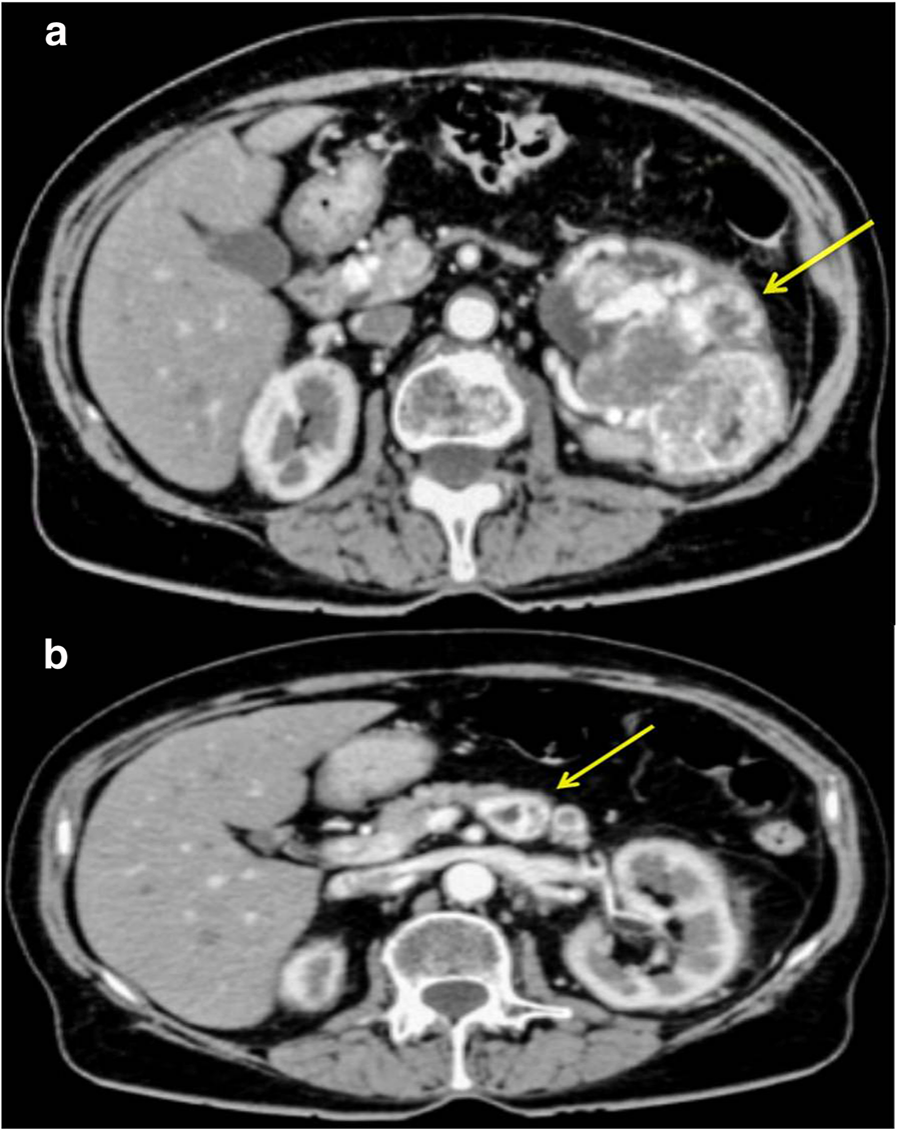
Pre-operative Computed Tomography (CT) scan: yellow arrows indicate the left renal mass (**a**) and its pancreatic metastasis (**b**)

A 68-year-old woman was admitted at our facility for gross haematuria and ultrasound scan positive for a left renal mass. After further evaluation with CT scan, a 13 cm mass (Fig. 1a) of left kidney (PADUA score 12), with a single pancreatic mass of about 2.5 cm, located in the pancreatic body, close to its tail were demonstrated (Fig. 1b). Work-up of staging ruled out other distant metastases or primary tumor, there was no evidence of inferior vena cava thrombosis and urinary cytology shows no abnormal cell. The patient referred no additional urological symptoms at the hospital intake. No major comorbidities were recorded: the Charlson Index score was 2, and the Eastern Cooperative Oncology Group (ECOG) was 1.

**Fig. 2:**
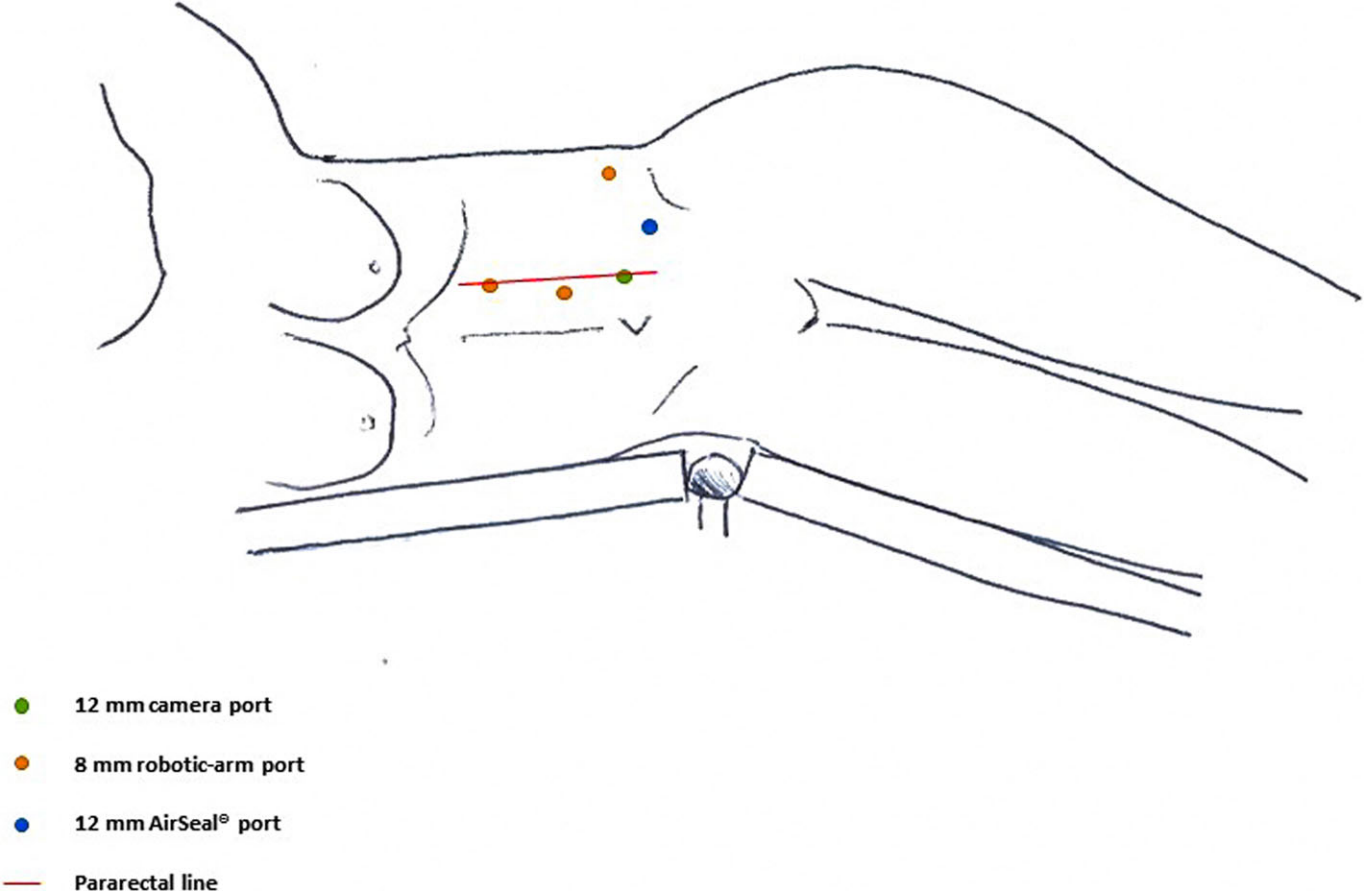
Robotic trocars’ positioning in lazy right lateral decubitus, angled at 45 degrees

**Fig. 3:**
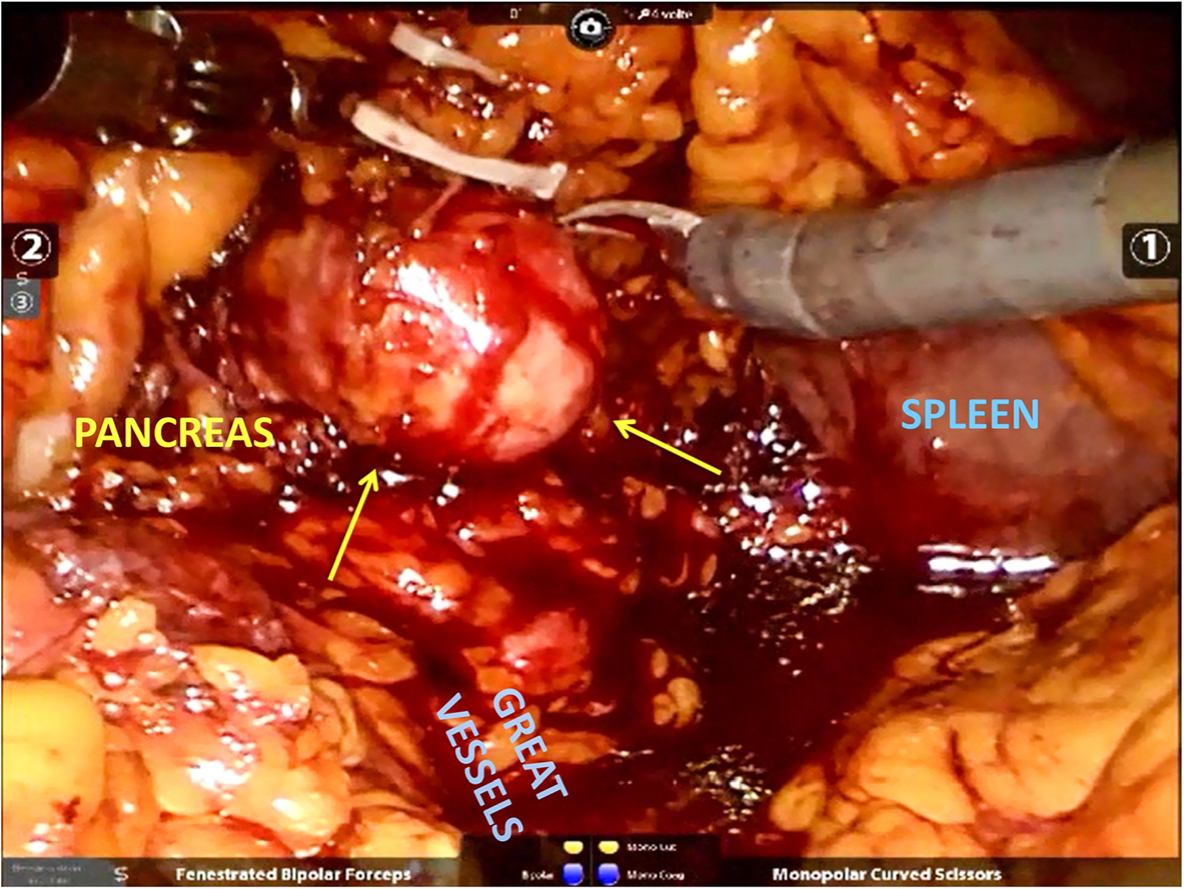
Intra-operative image shows the robotic dissection of metastatic lesion (yellow arrows) within the pancreatic tail, with preservation of splenic vessels

After tracheal intubation, under general anesthesia, the robot operating arms were installed behind the patient’s head. The procedure was entirely performed by a robotic-skilled urologist, with a general surgeon as bed-assistant, using the da Vinci Si® surgical system (Intuitive Surgical, Inc., Sunnyvale, CA, USA). We chose a trans-peritoneal approach, using a 5-port method, with lazy right lateral decubitus, angled at 45 degrees. Ports were placed in our usual robot assisted trans-peritoneal nephrectomy template, but they were shifted medially to accommodate for the planned distal pancreatectomy (Fig. 2). The optical trocar (12-mm) was placed at the umbilicus to allow the passage of a 30-degree and dual lens robotic camera. Three 8-mm robotic trocars were inserted for EndoWrists. CO_2_ pressure up to 12 mmHg was established. We started with a latero-colic incision and the dissection of the gastro-colic ligament. We entered into the epiploic retrocavity; the stomach was lifted up and the colon moved down by gravity. For better exposure of the pancreas’ tail, the transverse colon was freed up off its inferior border. We identified the body of the pancreas and the splenic vessels which were carefully dissociated by the pancreatic tail (Fig. 3). After that, we dissected the upper and lower edges of the normal pancreatic tissue, starting at the right side of the mass, in a latero-medial fashion. Through a bipolar dissection we isolated the metastasis using Hem-o-lok to ensure hemostasis. The dissection was conducted by closely controlling each parasitic vessel. Blunt dissection was applied when the tumour was close to the main pancreatic duct. The tumour was progressively mobilized from deep to superficial. Once the metastasectomy was completed we apposed Floseal® (Baxter Healthcare Corporation, Deerfield, Illinois, US) on the resection bed and the specimen was temporarily placed into an endo-bag. Then we began the renal dissection. Once the anterior surface of the kidney was exposed, multiple veins were encountered on the surface of Gerota’s fascia and controlled using individual Hem-o-lok. The renal hilum was completely dissected, being as medial as possible to ensure a good number of lymph node removals. Thus, we completed the left radical nephrectomy after division of ureter and gonadal vessels. No intra-operative complications were encountered. After positioning of both the specimens into the endo-bag we extracted them by peri-umbilical incision. A Jackson-Pratt drain was kept for 1 week.

**Fig. 4:**
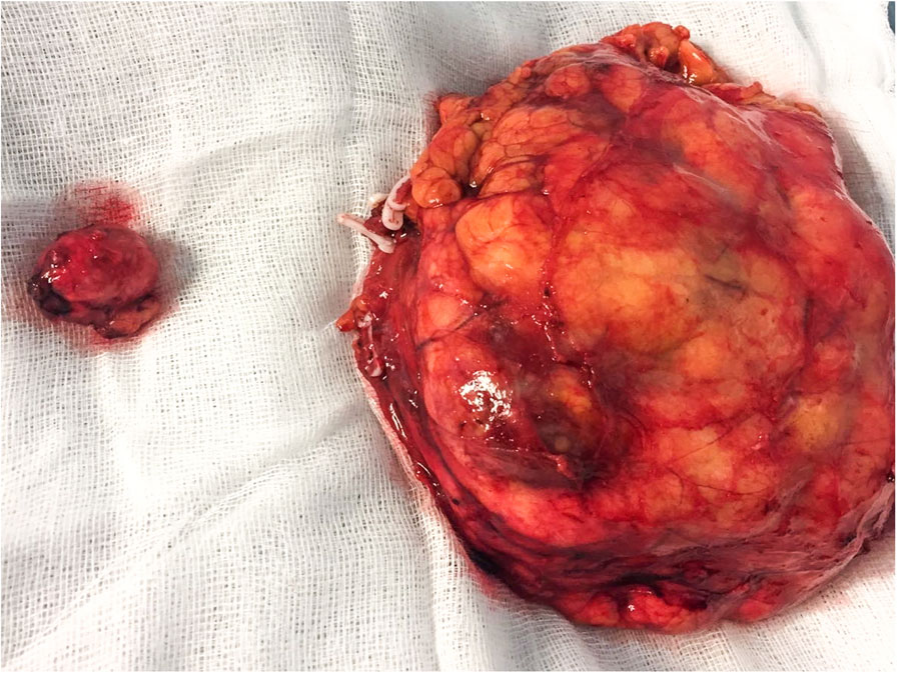
Specimen of left renal kidney and its pancreatic metastasis

**Fig. 5:**
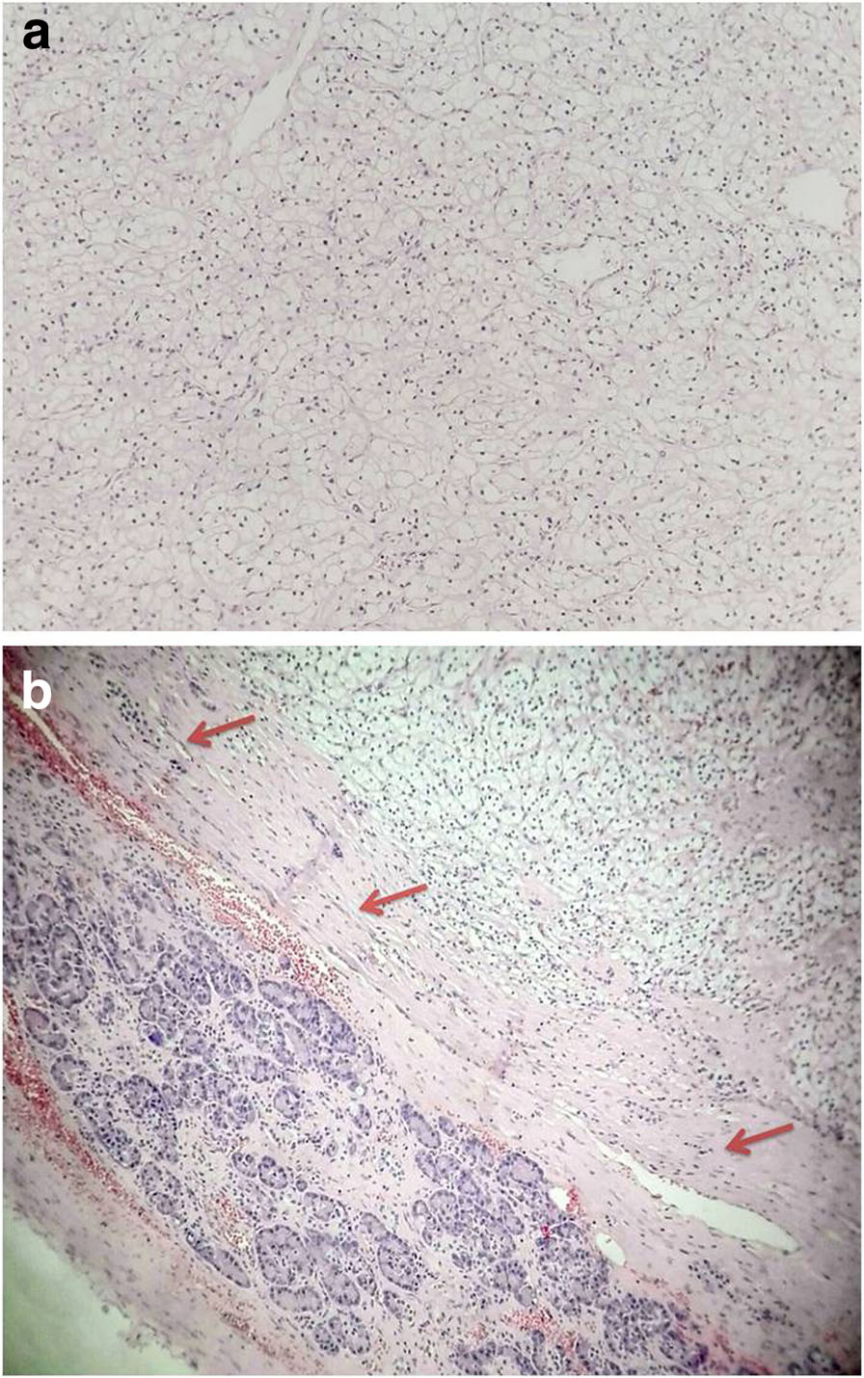
Microscopic evaluation of primary kidney cancer shows the histological appearance of RCC Fuhrman grade II (**a**). Microscopic evaluation of the metastasis specimen shows the histological appearance of RCC Fuhrman grade II, surrounded by its pseudocapsule (red arrows), which is partially covered by sane pancreatic parenchyma (**b**)

Peri-operative hemoglobin change was 2.4 g/dL (11.8–9.4 g/dL). Total operative time was 213 min and console time was 180 min. Postoperative total platelet count was 230.000/mmc. The post-operative course was uneventful. The patient was discharged at the 7th post-operative day, after drain removal. The gross examination shows a 13 cm encapsulated, yellowish-red mass of the left kidney, and a 2.5 cm enucleated pancreatic mass with similar visual characteristics (Fig. 4). The pathologic assessment showed a pT2b N0 M1 RCC of the left kidney, and a RCC metastasis in the body of the pancreas, both showing a Fuhrman grade II (5a-b). Pancreatic metastasis showed a fibrous avascular, pseudocapsular reaction surrounding malignant cell, as the primary RCC (Fig. 5b). Surgical margins were negative in both specimens. Serum creatinine at 1 month was 1.33 mg/dl. After 9 months of follow up the patient had no evidence of disease recurrence at whole-body TC scan. Thus, after multidisciplinary evaluation involving a urologist and medical oncologist no adjuvant therapy has yet to be administered.

## Discussion and conclusions

RCC represents the most common primary tumour leading to pancreatic metastasis, although the pancreas is only the fifth most frequent organ to be involved [5, 6, 17]. The incidence of synchronous disease is reported to be about 12% and, if pancreas is an isolated site of RCC disease it is associated with a more favourable prognosis compared to other metastatic sites [18]. While the removal of pancreatic metastases from other than RCC usually portends a poor prognosis, evidence is mounting that resection of RCC’s metastases is associated with improved outcomes [5, 20].

Minimally invasive surgery has become the gold standard in different common surgical procedures though pancreatic surgeons use this technique less frequently in their performances, despite the fact that robotic instruments give invaluable advantages over the laparoscopic approach. Here we present the first case of synchronous robotic nephrectomy plus enucleation of its pancreatic metastasis with spleen preservation.

A systematic bibliographic research up to March 2018 was conducted in PubMed and Scopus. The Preferred Reporting Items for Systematic Reviews and Meta-analyses (PRISMA) was followed for our bibliographic research (Additional file 1) [21]. Two authors (AB, DC) independently performed online bibliographic searches in order to identify titles and abstracts of interest.

The following search strategy were used in PubMed ((“pancreatectomy”[MeSH Terms] OR “pancreatectomy”[All Fields]) AND (“neoplasm metastasis”[MeSH Terms] OR (“neoplasm”[All Fields] AND “metastasis”[All Fields]) OR “neoplasm metastasis”[All Fields] OR “metastasis”[All Fields])) AND (“kidney neoplasms”[MeSH Terms] OR (“kidney”[All Fields] AND “neoplasms”[All Fields]) OR “kidney neoplasms”[All Fields] OR (“renal”[All Fields] AND “cancer”[All Fields]) OR “renal cancer”[All Fields] OR “nephrectomy”[All Fields]).

All titles and abstracts were assessed to select those focusing on pancreatic conservative surgery for RCC metastasis. Subsequently, the full-text of the selected papers were independently screened by two authors (AB and GC) for eligibility. When there was overlapping between multiple articles published by the same authors and no difference in the examined time, only the most recent paper was enclosed to avoid double counting. The Pubmed function “related articles” and Scopus database were used to search further articles.

In this review, we considered both comparative and non-comparative studies, irrespectively of their size, publication status and language, which included patients who underwent conservative pancreatic surgery for RCC metastasis, irrespectively of the type of surgical approach used for comparative group (robotic, laparoscopic or open).

Studies which not reported conservative pancreatic surgery for metastasis originating from RCC were excluded.

Full texts of relevant articles were further assessed for inclusion in this study. We finally included 32 articles (Table 1).

**Table 1:** Review of previous reported case of surgical treatment of RCC pancreatic metastases

Reference	Year	N° of cases	Mean age (yy)	% Female (N)	Histology	Fuhrman	Mean metastases size (cm)	Surgical approach	Operative procedure (n)	% Synchronous with primary (N)	Post-operative complications	Median follow-up after metastasectomy (months)
Yagi et al. [52]	2017	7	59	57% (4)	Clear cells	NA	2	Open	DP (4) + PPPD (2) + TP (1)	0	Fistula (1)	138
Nihei et al. [53]	2016	1	69	100% (1)	Clear cells	NA	2	Open	DP + splenectomy	0	0	228
Miura et al. [54]	2016	1	72	0	Clear cells	2	NA	Open	STP	0	0	20
Abdul-Muhsin et al. [16]	2016	1	57	100 (1)	Clear cells	III	3	Robot-assisted	Left nefrectomy + DP + splenectomy (1)	100 (1)	0	12
Boussios et al. [55]	2016	1	63	0	NA	II	1.5	Open	DP + splenectomy + cholecystectomy (1)	0	0	6
Garcia-Mayor FernàNAez et al. [56]	2016	1	72	100 (1)	NA	NA	NA	Open	DP + splenectomy (1)	0	NA	NA
Facy et al. [57]	2013	13	65	46 (6)	NA	NA	NA	Open	NA	8.3 (1)	NA	48
Niess et al. [58]	2013	16	65	50 (8)	NA	NA	3.1	Open	DP + splenectomy (7); DP (3); TP (1); PPPD (3); WPD (2)	NA	NA	NA
Zygulska et al. [59]	2012	1	76	100 (1)	NA	NA	NA	Open	DP + splenectomy (1)	0	NA	NA
Huscher et al. [22]	2012	1	67	100 (1)	NA	NA	Locally advanced	Laparoscopy	Left nefrectomy + DP + splenectomy (1)	/	NA	NA
Yazbek et al. [31]	2012	14	73	9.1 (1)	NA	NA	NA	Open	3 WPD, 4 DP with spleen-preservation, 1 Completion Pancreatectomy, 4 enucleations aNA 2 enucleo-resections	9.1 (1)	36.4 (4)	NA
Thadani et al. [60]	2011	1	67	100 (1)	Clear cells	NA	5.8	Open	DP + splenectomy (1)	0	NA	NA
You et al. [61]	2011	7	NA	NA	NA	NA	NA	Open	NA	0	NA	NA
Barbaros et al. [24]	2010	1	59	100 (1)	Clear cells	NA	3 + 1.5	Single- site laparoscopy	DP + splenectomy (1)	0	100 (1)	NA
Konstantinidis et al. [35]	2010	20	68	35 (7)	NA	NA	3	Open	NA	5 (1)	NA	36
Mourra et al. [62]	2010	8	NA	NA	NA	NA	NA	Open	NA	0	NA	NA
Strobel et al. [63]	2009	31	NA	NA	NA	NA	3	Open	NA	0	NA	NA
Reddy et al. [34]	2008	21	NA	NA	NA	NA	4	Open	NA	NA	NA	NA
Zerbi et al. [33]	2008	23	65	31 (7)	NA	NA	NA	Open	NA	0	39.1 (9)	31
Eidt et al. [20]	2007	7	NA	NA	NA	NA	4.9	Open	DP + splenectomy (1); TP (3); PPPD (2)	NA	NA	46
Crippa et al. [64]	2006	5	64	60 (3)	NA	NA	NA	Open	DP + splenectomy (3); PPPD (1); WPD (1)	0	NA	NA
Jarufe et al. [65]	2005	7	NA	NA	NA	NA	NA	Open	NA	NA	NA	NA
De Fazio et al. [66]	2004	1	74	0	NA	NA	NA	Open	DP + splenectomy (1)	0	100 (1)	NA
Moussa et al. [67]	2004	7	NA	NA	NA	NA	NA	Open	NA	NA	0	NA
Bassi et al. [36]	2003	17	64	32 (5)	NA	NA	NA	Open	7 DP with splenectomy, 2 PDs, 2 TPs 3 DPPHR, 1 MD aNA two enucleations, one of which was carried out in combination with a DP)	0	47.1 (8)	33
Giulini et al. [48]	2003	1	73	100 (1)	Clear cells	NA	NA	Open	Metastatectomy (1)	0	NA	NA
Hernanez et al. [68]	2003	1	64	0	Clear cells	NA	2	Laparoscopy	DP (1)	0	0	NA
Law et al. [69]	2003	14	64	64 (9)	NA	NA	NA	Open	NA	7.7 (1)	0	32
Yachida et al. [70]	2002	1	66	0	NA	NA	2,5	Open	DP + splenectomy (1)	0	0	NA
Fricke et al. [71]	2000	1	69	100 (1)	NA	NA	NA	Open	DP + splenectomy (1)	0	NA	NA
Ghavamian et al. [19]	2000	11	66	66 (7)	NA	NA	NA	Open	NA	0	0	48
Le Borgne et al. [72]	2000	5	NA	NA	NA	NA	NA	Open	NA	NA	NA	NA

Abbreviations: *NA* not available, *DP* distal pancreatectomy, *STP* sub-total pancreatectomy, *TP* total pancreatectomy, *MD* middle pancreatectomy, *PPPD* pylorus preserving pancreaticoduodenectomy, *WPD* whipple pancreaticoduodenectomy, *DPPHR* duodenum-preserving pancreatic head resection

Three cases of laparoscopic distal pancreatectomy for metastatic RCC (mRCC) were reported [22–24]. A unique case of single site distal pancreatectomy and splenectomy was performed [24]. In one case “en bloc” removal of distal pancreas, left kidney and spleen was performed [16]. A single case of robotic “en bloc” resection was only recently reported [18]. Recently, McNichols et al. found that among the 158 patients with RCC who survived more than 10 years, 11% had late recurrence in the form of metastasis [25]. Typically, metastasis is diagnosed many years after nephrectomy, with a longer time to metastatic disease associated with better prognosis, reflecting a relatively indolent disease [26, 27]. The five-year survival rate of patients with untreated metastatic renal cell carcinoma is account to be of 13%, while it grows up to 65% after surgical resection [28, 29].

In large studies, most of pancreatic metastasectomies are performed using a standard pancreatic resection, that includes either Pancreatico-Duodenectomy (PD), or Distal Pancreatectomy (DP), or Total Pancreatectomy (TP) [26]. Among the three known types of pancreatic involvement by RCC, the most common (50–73%) is that of a solitary, well-defined mass, rather than multiple pancreatic lesions (5–10%) and diffused metastatic infiltration causing enlargement of the organ (15–44%) [30]. Atypical resection for RCC metastasis, such as enucleation, enucleoresection or central pancreatectomy, seems to be associated with better quality of life without diabetes mellitus by preserving a maximum of pancreatic tissue [31]. However, their role is less studied, and this approaches is reserved to multilocality [7].

Considering both minimally-invasive and open approaches, the surgically removed RCC metastasis’ range of size is reported to be within 1.5 and 4.9 cm, (Table 1). However, the size of the tumor is not the main factor determining the type of resection, whereas the depth in organ involvement is of high importance, with a distance > 3 mm from the main pancreatic duct consider as safe to proceed with pancreatic enucleation [32]. One of the arguments supporting standard resection instead of an atypical one is the ability to find pancreatic lymph nodes; although an extensive review of the literature indicates that the involvement of lymph nodes in metastatic pancreatic malignancy is extremely unusual, not affecting the patient’s prognosis [18, 33, 34]. Another argument against atypical resection is the high early recurrency rate, reported by Bassi et al. to be about 50%. Zerbi did not confirmed these results and proposed that this high recurrent rate was determined by undetected multilocality rather than as the consequence of an inadequate surgical procedure [31, 33, 35]. In our opinion, the high recurrency rate could be partially explained by the absence of modern immunotherapies and diagnostic tools at the time of these studies [36].

Organ-sparing treatment of pancreatic metastasis seems to be unexceptionable thanks to a similar fibrous avascular, pseudocapsular reaction that surrounds the tumour as previously demonstrated [36–38]. In particular, robotic tumor enucleation was judged as safe and effective for benign or borderline tumors in both sides of the pancreas and did not increases the rate of clinical major complications, as comparing to the open approach [39]. Our pathological report confirms similar characteristics between the pancreatic metastasis and the primary RCC (Fig. 5a –b).

Beyond the introduction of new surgical techniques, the management of mRCC has changed dramatically in the last 20 years, thanks to the development of effective immunotherapies for advanced disease [6, 11, 12]. The major change with reference to treatment for mRCC was the introduction of drugs directed against the Vascular Endothelial Growth Factor (VEGF) and mammalian Target Of Rapamycin (mTOR) pathway. In addition, the high rate of responses obtained by the use of Tyrosine Kinase Inhibitors (TKIs) in this subpopulation, suggest their use as neo-adjuvant or adjuvant therapies, even though the median survival of patients undergoing surgery was reported to be 103 months versus 86 months in patients treated with TKIs [27].

Not by chance, in a metastatic kidney disease the resection of primary tumour combined with adjuvant immunotherapy is justified by the improved prognosis, due to an enhanced immune-mediate response to systemic treatment and removal of a source of growth factors and immunosuppressive molecules. A patient obtains a benefit from a metastasectomy only when the primary tumour is resected, not only because of relief from mass-related pain or haematuria, but also for removal of a source of additional metastases and para-neoplastic syndrome [40–42].

Validated prognostic factors are needed to choose the best management of these patients and the best cost-effectiveness strategy because of the wide range of low- and high-grade adverse effects linked to the use of the TKIs [27]. In fact, since the introduction of the Memorial Sloan–Kettering Cancer Center (MSKCC) three risk categories, it was clear that the response to systemic therapies is mainly linked to patients’ clinical and laboratory parameters [28]. In addition, the International Kidney Cancer Working Group identified five independent prognostic variables (haemoglobin, white cell count, LDH, alkaline phosphatase and calcium) [6]. The removal of the spleen may affects these parameters while a spleen-sparing procedure maintains the platelet count, preserving post-operative immunologic capabilities [43–46]. This conservative surgery was performed, to date, mainly for benign tumours or low-grade malignancies of the body and the tail of pancreas or for chronic pancreatitis [47]. Giulini et al. reported a case of pancreas metastasectomy with spleen preservation for a 2.6 cm pancreatic mass diagnosed 24 years after nephrectomy [48]. Robot-assisted surgery allow a meticulous control of the splenic vessel fundamentals for its preservation [15]. Moreover, a robotic approach is linked to a better splenic preservation and lower positive margins rate, a minor hospital stay, and a better and faster recovery, as demonstrated by a recent meta-analysis [49].

Neverthless, as first step our patient was advised on a considerable chance of conversion to open surgery. We decided to perform a robotic approach followed, eventually, by a post-operative immunotherapy [42, 50]. It should be noted that this robotic procedure is complex and the surgical indication should be carefully examined. The surgeon should be prepared for open conversion and vascular complications [16]. We believe that in selected patients, pancreatic metastasectomy is safe and improves overall survival. However a cautious approach should be adopted taking into consideration the biological behaviour of the primary tumour given as the morbidity of pancreatic surgery varies between 20 to 40% [51]. In our opinion, the preservation of the spleen in the case of synchronous resection of primary and metastatic tumour can be of paramount importance in consideration of the necessity of adjuvant systemic treatment [44]. Future research in biological features associated with tumor behavior and tumor response to therapy are needed to determine the best strategies for an individualized therapeutic approach.

## Additional file

Additional file 1: PRISMA flow chart of literature search. We report a schematic resume of our bibliographic research strategy in order to select paper focusing on pancreatic conservative surgery for RCC metastasis, according to PRISMA guidelines. (PDF 107 kb)

## Abbreviations

RCC: Renal cell carcinoma
mRCC: Metastatic renal cell carcinoma
PD: Pancreatico-Duodenectomy
DP: Distal pancreatectomy
TP: Total pancreatectomy
ECOG: Eastern Cooperative Oncology Group
MSKCC: Memorial Sloan–Kettering Cancer Center
VEGF: Vascular endothelial growth factor
mTOR: Mammalian Target Of Rapamycin
TKIs: Tyrosine kinase inhibitors

## References

[1] Jemal A, Siegel R, Xu J, Ward E (2010). Cancer statistics, 2010. CA Cancer J Clin.

[2] Sohn TA, Yeo CJ, Cameron JL, Nakeeb A, Lillemoe KD (2001). Renal cell carcinoma metastatic to the pancreas: results of surgical management. J Gastrointest Surg.

[3] Karellas ME, Jang TL, Kagiwada MA, Kinnaman MD, Jarnagin WR, Russo P (2009). Advanced-stage renal cell carcinoma treated by radical nephrectomy and adjacent organ or structure resection. BJU Int.

[4] Humphrey PA, Moch H, Cubilla AL, Ulbright TM, Reuter VE (2016). The 2016 WHO classification of Tumours of the urinary system and male genital organs-part B: prostate and bladder Tumours. Eur Urol.

[5] Thomas AZ, Adibi M, Borregales LD, Wood CG, Karam JA (2015). Role of metastasectomy in metastatic renal cell carcinoma. Curr Opin Urol.

[6] Manola J, Royston P, Elson P, McCormack JB, Mazumdar M, Negrier S, Escudier B, Eisen T, Dutcher J, Atkins M (2011). Prognostic model for survival in patients with metastatic renal cell carcinoma: results from the international kidney cancer working group. Clin Cancer Res.

[7] Reddy S, Wolfgang CL (2009). The role of surgery in the management of isolated metastases to the pancreas. Lancet Oncol.

[8] Chua TC, Petrushnko W, Mittal A, Gill AJ, Samra JS (2016). Pancreatic Metastasectomy-an analysis of survival outcomes and prognostic factors. J Gastrointest Surg.

[9] Marudanayagam R, Ramkumar K, Shanmugam V, Langman G, Rajesh P, Coldham C, Bramhall SR, Mayer D, Buckels J, Mirza DF (2009). Long-term outcome after sequential resections of liver and lung metastases from colorectal carcinoma. HPB (Oxford).

[10] Garden OJ, Rees M, Poston GJ, Mirza D, Saunders M, Ledermann J, Primrose JN, Parks RW (2006). Guidelines for resection of colorectal cancer liver metastases. Gut.

[11] Lam JS, Shvarts O, Pantuck AJ (2004). Changing concepts in the surgical management of renal cell carcinoma. Eur Urol.

[12] Husillos Alonso A, Carbonero Garcia M, Gonzalez Enguita C (2015). Is there a role for systemic targeted therapy after surgical treatment for metastases of renal cell carcinoma?. World J Nephrol.

[13] Hiotis SP, Klimstra DS, Conlon KC, Brennan MF (2002). Results after pancreatic resection for metastatic lesions. Ann Surg Oncol.

[14] Wright GP, Zureikat AH (2016). Development of minimally invasive pancreatic surgery: an evidence-based systematic review of laparoscopic versus robotic approaches. J Gastrointest Surg.

[15] Parisi A, Coratti F, Cirocchi R, Grassi V, Desiderio J, Farinacci F, Ricci F, Adamenko O, Economou AI, Cacurri A (2014). Robotic distal pancreatectomy with or without preservation of spleen: a technical note. World J Surg Oncol.

[16] Abdul-Muhsin HM, Stern KL, Katariya NN, Castle EP (2016). Robot assisted “en bloc” radical nephrectomy, splenectomy and distal pancreatectomy for renal cell carcinoma: case report and illustration of technique. J Robot Surg.

[17] Adsay NV, Andea A, Basturk O, Kilinc N, Nassar H, Cheng JD (2004). Secondary tumors of the pancreas: an analysis of a surgical and autopsy database and review of the literature. Virchows Arch.

[18] Sellner F, Tykalsky N, De Santis M, Pont J, Klimpfinger M (2006). Solitary and multiple isolated metastases of clear cell renal carcinoma to the pancreas: an indication for pancreatic surgery. Ann Surg Oncol.

[19] Ghavamian R, Klein KA, Stephens DH, Welch TJ, LeRoy AJ, Richardson RL, Burch PA, Zincke H (2000). Renal cell carcinoma metastatic to the pancreas: clinical and radiological features. Mayo Clin Proc.

[20] Eidt S, Jergas M, Schmidt R, Siedek M (2007). Metastasis to the pancreas--an indication for pancreatic resection?. Langenbecks Arch Surg.

[21] Moher D, Shamseer L, Clarke M, Ghersi D, Liberati A, Petticrew M, Shekelle P, Stewart LA, Group P-P (2015). Preferred reporting items for systematic review and meta-analysis protocols (PRISMA-P) 2015 statement. Syst Rev.

[22] Huscher CG, Mingoli A, Sgarzini G, Mereu A (2012). Laparoscopic left nephrectomy with “en bloc” distal splenopancreatectomy. Ann Surg Oncol.

[23] Hernandez F, Rha KH, Pinto PA, Kim FJ, Klicos N, Chan TY, Kavoussi L, Jarrett TW (2003). Laparoscopic nephrectomy: assessment of morcellation versus intact specimen extraction on postoperative status. J Urol.

[24] Barbaros U, Sumer A, Demirel T, Karakullukcu N, Batman B, Icscan Y, Saricam G, Serin K, Loh WL, Dinccag A (2010). Single incision laparoscopic pancreas resection for pancreatic metastasis of renal cell carcinoma. JSLS.

[25] McNichols DW, Segura JW, DeWeerd JH (1981). Renal cell carcinoma: long-term survival and late recurrence. J Urol.

[26] Wente MN, Kleeff J, Esposito I, Hartel M, Muller MW, Frohlich BE, Buchler MW, Friess H (2005). Renal cancer cell metastasis into the pancreas: a single-center experience and overview of the literature. Pancreas.

[27] Santoni M, Conti A, Partelli S, Porta C, Sternberg CN, Procopio G, Bracarda S, Basso U, De Giorgi U, Derosa L (2015). Surgical resection does not improve survival in patients with renal metastases to the pancreas in the era of tyrosine kinase inhibitors. Ann Surg Oncol.

[28] Motzer RJ, Mazumdar M, Bacik J, Berg W, Amsterdam A, Ferrara J (1999). Survival and prognostic stratification of 670 patients with advanced renal cell carcinoma. J Clin Oncol.

[29] Sweeney AD, Fisher WE, Wu MF, Hilsenbeck SG, Brunicardi FC (2010). Value of pancreatic resection for cancer metastatic to the pancreas. J Surg Res.

[30] Ballarin R, Spaggiari M, Cautero N, De Ruvo N, Montalti R, Longo C, Pecchi A, Giacobazzi P, De Marco G, D'Amico G (2011). Pancreatic metastases from renal cell carcinoma: the state of the art. World J Gastroenterol.

[31] Yazbek T, Gayet B (2012). The place of enucleation and enucleo-resection in the treatment of pancreatic metastasis of renal cell carcinoma. JOP.

[32] Huttner FJ, Koessler-Ebs J, Hackert T, Ulrich A, Buchler MW, Diener MK (2015). Meta-analysis of surgical outcome after enucleation versus standard resection for pancreatic neoplasms. Br J Surg.

[33] Zerbi A, Ortolano E, Balzano G, Borri A, Beneduce AA, Di Carlo V (2008). Pancreatic metastasis from renal cell carcinoma: which patients benefit from surgical resection?. Ann Surg Oncol.

[34] Reddy S, Edil BH, Cameron JL, Pawlik TM, Herman JM, Gilson MM, Campbell KA, Schulick RD, Ahuja N, Wolfgang CL (2008). Pancreatic resection of isolated metastases from nonpancreatic primary cancers. Ann Surg Oncol.

[35] Konstantinidis IT, Dursun A, Zheng H, Wargo JA, Thayer SP, Fernandez-del Castillo C, Warshaw AL, Ferrone CR (2010). Metastatic tumors in the pancreas in the modern era. J Am Coll Surg.

[36] Bassi C, Butturini G, Falconi M, Sargenti M, Mantovani W, Pederzoli P (2003). High recurrence rate after atypical resection for pancreatic metastases from renal cell carcinoma. Br J Surg.

[37] Ficarra V, Galfano A, Cavalleri S (2009). Is simple enucleation a minimal partial nephrectomy responding to the EAU guidelines’ recommendations?. Eur Urol.

[38] Pansadoro A, Cochetti G, D'Amico F, Barillaro F, Del Zingaro M, Mearini E (2015). Retroperitoneal laparoscopic renal tumour enucleation with local hypotension on demand. World J Urol.

[39] Jin JB, Qin K, Li H, Wu ZC, Zhan Q, Deng XX, Chen H, Shen BY, Peng CH, Li HW (2016). Robotic enucleation for benign or borderline Tumours of the pancreas: a retrospective analysis and comparison from a high-volume Centre in Asia. World J Surg.

[40] Polcari AJ, Gorbonos A, Milner JE, Flanigan RC (2009). The role of cytoreductive nephrectomy in the era of molecular targeted therapy. Int J Urol.

[41] Flanigan RC, Yonover PM (2001). The role of radical nephrectomy in metastatic renal cell carcinoma. Semin Urol Oncol.

[42] Flanigan RC, Mickisch G, Sylvester R, Tangen C, Van Poppel H, Crawford ED (2004). Cytoreductive nephrectomy in patients with metastatic renal cancer: a combined analysis. J Urol.

[43] Weledji EP (2014). Benefits and risks of splenectomy. Int J Surg.

[44] Kwon W, Jang JY, Kim JH, Chang YR, Jung W, Kang MJ, Kim SW (2016). An analysis of complications, quality of life, and nutritional index after laparoscopic distal pancreatectomy with regard to spleen preservation. J Laparoendosc Adv Surg Tech A.

[45] Francke EL, Neu HC (1981). Postsplenectomy infection. Surg Clin North Am.

[46] Sugimachi K, Kodama Y, Kumashiro R, Kanematsu T, Noda S, Inokuchi K (1980). Critical evaluation of prophylactic splenectomy in total gastrectomy for the stomach cancer. Gan.

[47] Kimura W, Yano M, Sugawara S, Okazaki S, Sato T, Moriya T, Watanabe T, Fujimoto H, Tezuka K, Takeshita A (2010). Spleen-preserving distal pancreatectomy with conservation of the splenic artery and vein: techniques and its significance. J Hepatobiliary Pancreat Sci.

[48] Giulini SM, Portolani N, Bonardelli S, Baiocchi GL, Zampatti M, Coniglio A, Baronchelli C (2003). Distal pancreatic resection with splenic preservation for metastasis of renal carcinoma diagnosed 24 years later from the nephrectomy. Ann Ital Chir.

[49] Guerrini GP, Lauretta A, Belluco C, Olivieri M, Forlin M, Basso S, Breda B, Bertola G, Di Benedetto F (2017). Robotic versus laparoscopic distal pancreatectomy: an up-to-date meta-analysis. BMC Surg.

[50] Flanigan RC, Salmon SE, Blumenstein BA, Bearman SI, Roy V, McGrath PC, Caton JR, Munshi N, Crawford ED (2001). Nephrectomy followed by interferon alfa-2b compared with interferon alfa-2b alone for metastatic renal-cell cancer. N Engl J Med.

[51] Adler H, Redmond CE, Heneghan HM, Swan N, Maguire D, Traynor O, Hoti E, Geoghegan JG, Conlon KC (2014). Pancreatectomy for metastatic disease: a systematic review. Eur J Surg Oncol.

[52] Yagi T, Hashimoto D, Taki K, Yamamura K, Chikamoto A, Ohmuraya M, Beppu T, Baba H (2017). Surgery for metastatic tumors of the pancreas. Surg Case Rep.

[53] Nihei K, Sakamoto K, Suzuki S, Mishina T, Otaki M (2016). A case of pancreatic metastasis of renal cell carcinoma. Gan To Kagaku Ryoho.

[54] Miura T, Nakamura N, Ogawa K, Watanabe Y, Yonekura K, Sanada T, Kuwabara H, Goseki N (2016). Resection of pancreatic metastasis from renal cell carcinoma 21 years after nephrectomy. Gan To Kagaku Ryoho.

[55] Boussios S, Zerdes I, Batsi O, Papakostas VP, Seraj E, Pentheroudakis G, Glantzounis GK (2016). Pancreatic resection for renal cell carcinoma metastasis: an exceptionally rare coexistence. Int J Surg Case Rep.

[56] Garcia-Mayor Fernandez RL, Fernandez-Gonzalez M. Diagnosis and treatment of isolated pancreatic metastases from renal clear cell carcinoma: report of a case and review of literature. Cirugia y Cirujanos. 2017;85(5):436–439. 10.1016/j.circir.2016.05.007 27417704

[57] Facy O, Angot C, Guiu B, Al Samman S, Matte A, Rat P, Ortega-Deballon P (2013). Interest of intraoperative ultrasonography during pancreatectomy for metastatic renal cell carcinoma. Clin Res Hepatol Gastroenterol.

[58] Niess H, Conrad C, Kleespies A, Haas F, Bao Q, Jauch KW, Graeb C, Bruns CJ (2013). Surgery for metastasis to the pancreas: is it safe and effective?. J Surg Oncol.

[59] Zygulska AL, Wojcik A, Richter P, Krzesiwo K (2012). Renal carcinoma metachronous metastases to the gall-bladder and pancreas--case report. Pol Przegl Chir.

[60] Thadani A, Pais S, Savino J (2011). Metastasis of renal cell carcinoma to the pancreas 13 years postnenhrectomv. Gastroenterol Hepatol.

[61] You DD, Choi DW, Choi SH, Heo JS, Kim WS, Ho CY, Lee HG (2011). Surgical resection of metastasis to the pancreas. J Korean Surg Soc.

[62] Mourra N, Arrive L, Balladur P, Flejou JF, Tiret E, Paye F (2010). Isolated metastatic tumors to the pancreas: Hopital St-Antoine experience. Pancreas.

[63] Strobel O, Buchler MW (2015). Pancreatic metastases from tumors in the urogenital tract. Gastrointest Tumors.

[64] Crippa S, Angelini C, Mussi C, Bonardi C, Romano F, Sartori P, Uggeri F, Bovo G (2006). Surgical treatment of metastatic tumors to the pancreas: a single center experience and review of the literature. World J Surg.

[65] Jarufe N, McMaster P, Mayer AD, Mirza DF, Buckels JA, Orug T, Tekin K, Bramhall SR (2005). Surgical treatment of metastases to the pancreas. Surgeon.

[66] De Fazio S, Destito C, Ricciardi V, Marin AW (2004). Pancreatic metastasis of renal cell carcinoma: a case report and review of the literature. Il Giornale di Chirurgia.

[67] Moussa A, Mitry E, Hammel P, Sauvanet A, Nassif T, Palazzo L, Malka D, Delchier JC, Buffet C, Chaussade S (2004). Pancreatic metastases: a multicentric study of 22 patients. Gastroenterologie Clinique et Biologique.

[68] Hernandez DJ, Kavoussi LR, Ellison LM (2003). Laparoscopic distal pancreatectomy for metastatic renal cell carcinoma. Urology.

[69] Law CH, Wei AC, Hanna SS, Al-Zahrani M, Taylor BR, Greig PD, Langer B, Gallinger S (2003). Pancreatic resection for metastatic renal cell carcinoma: presentation, treatment, and outcome. Ann Surg Oncol.

[70] Yachida S, Fukushima N, Kanai Y, Nimura S, Shimada K, Yamamoto J, Sakamoto M (2002). Pancreatic metastasis from renal cell carcinoma extending into the main pancreatic duct: a case report. Jpn J Clin Oncol.

[71] Fricke P, Schulz HU, Buhtz P, Lippert H (2000). Multiple metachronous metastases of renal cell carcinoma in the pancreas. Case report and review of the literature. Chirurg.

[72] Le Borgne J, Partensky C, Glemain P, Dupas B, de Kerviller B (2000). Pancreaticoduodenectomy for metastatic ampullary and pancreatic tumors. Hepato-Gastroenterology.

