# Supplementary figures and images for: Robotic treatment of oligometastatic kidney tumor with synchronous pancreatic metastasis: case report and review of the literature
Source: BMC Surg. 2018 Jun 13;18:40. doi: 10.1186/s12893-018-0371-x (PMC5998557; doi:10.1186/s12893-018-0371-x)

PRISMA flow chart of literature search

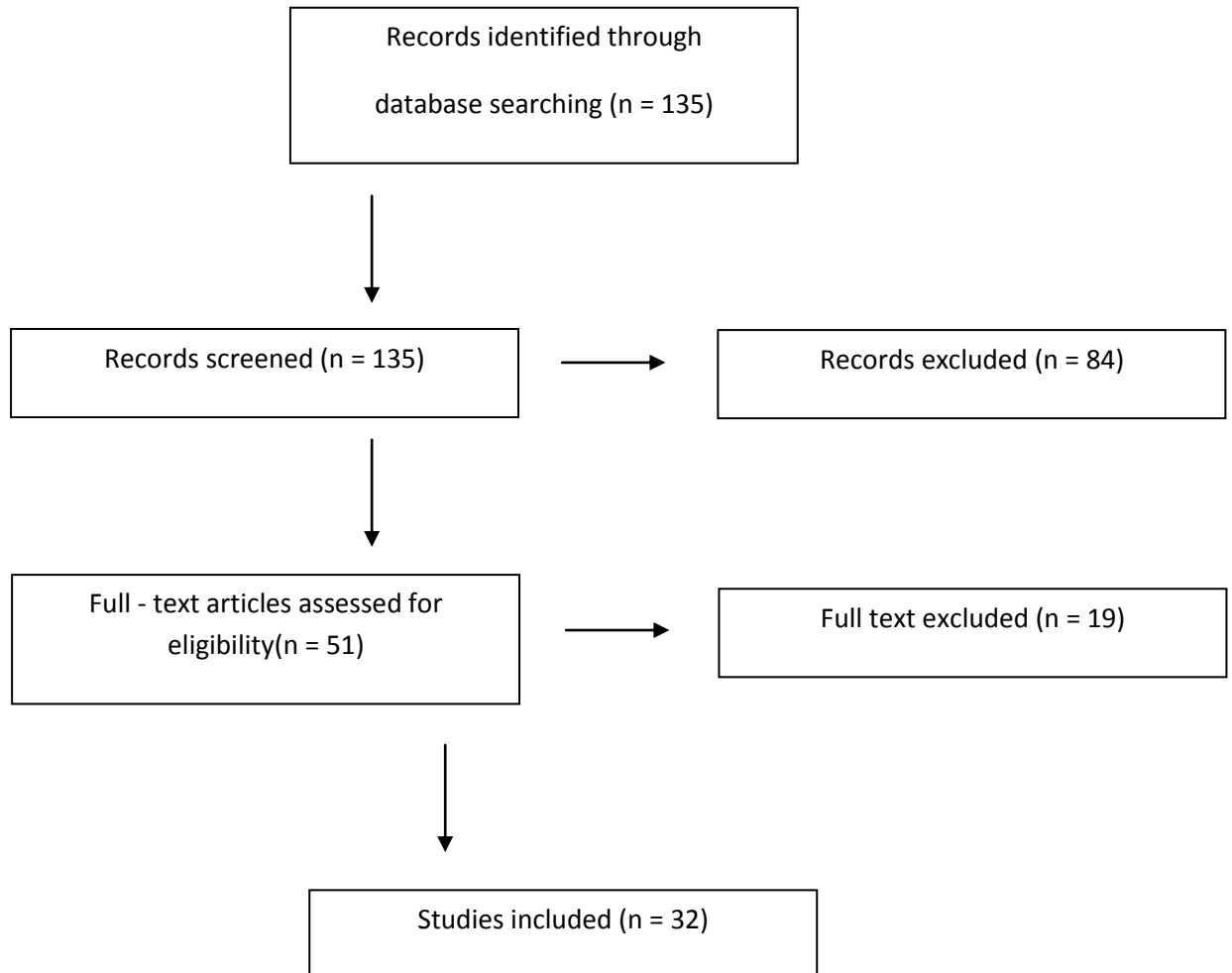

Supplement: Supplementary file 1 — PRISMA flow chart of literature search. We report a schematic resume of our bibliographic research strategy in order to select paper focusing on pancreatic conservative surgery for RCC metastasis, according to PRISMA guidelines. (PDF 107 kb) [file 12893_2018_371_MOESM1_ESM.pdf]
